# Supplementary material for: Calibration of cognitive tests to address the reliability paradox for decision-conflict tasks
Source: Nat Commun. 2023 Apr 19;14:2234. doi: 10.1038/s41467-023-37777-2 (PMC10115879; doi:10.1038/s41467-023-37777-2)
Supplement: Supplementary file 1 — Supplementary Information [file 41467_2023_37777_MOESM1_ESM.pdf]

Supplementary Information

Calibration of cognitive tests to address the reliability paradox for decision-conflict tasks

Talira Kucina, Lindsay Wells, Ian Lewis, Kristy de Salas, Amelia Kohl, Matt Palmer, James D. Sauer, Dora Matzke, Eugene Aidman, and Andrew Heathcote

Correspondence to: talira.kucina@utas.edu.au

Supplementary Analyses

Main Experiment

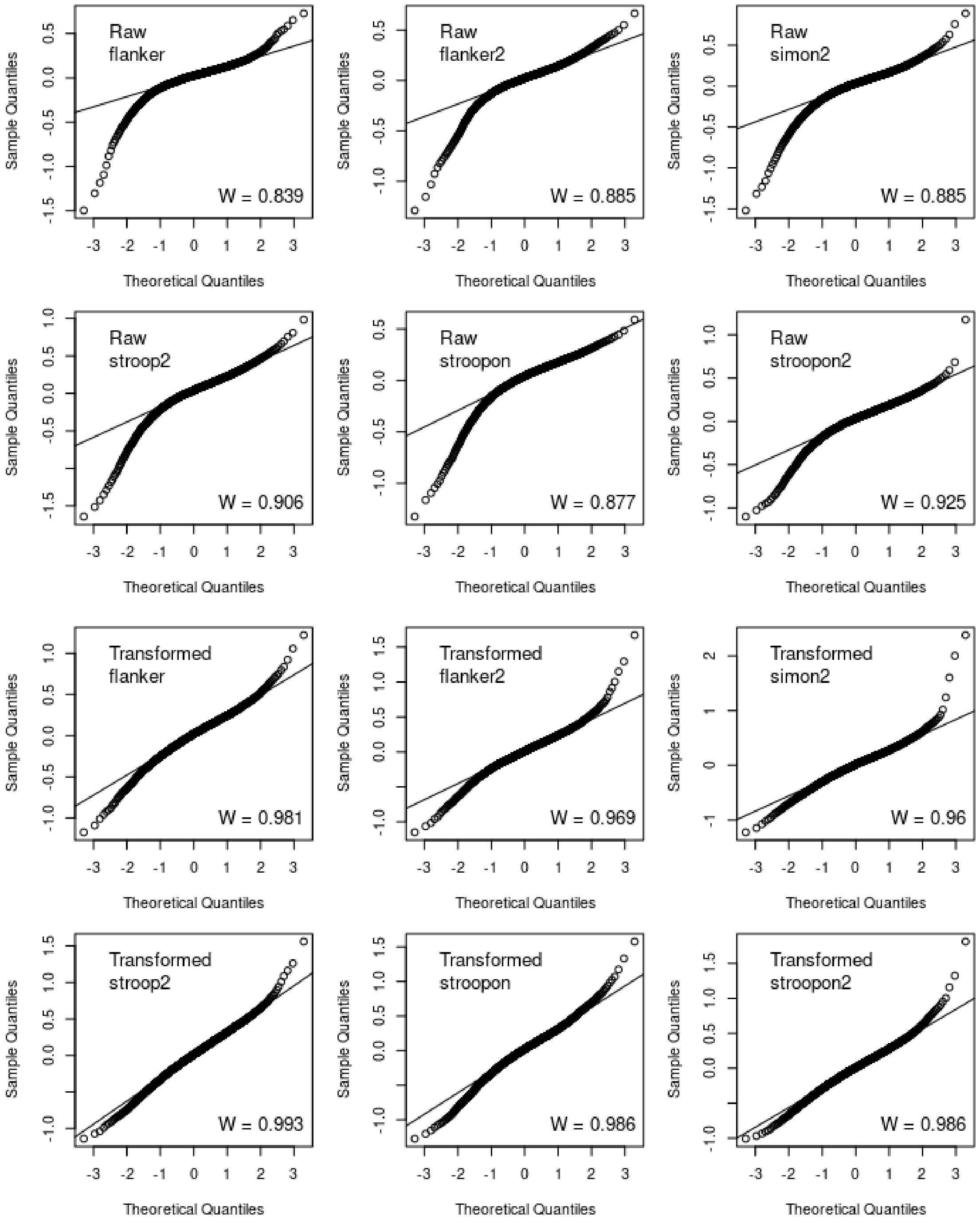

Supplementary Figure 1. Normality tests for the final gamified experiment. Q-Q plots and Shapiro-Wilk (*W*) values for each of the tasks. Top two panels: Raw (untransformed) response time data, bottom two panels: transformed ($\log_e$(RT-0.2)) response time data. The Q-Q plots on the transformed data indicate the data more closely fit a normal distribution. Combined with higher *W* values, this suggests that the transformation was effective. Based on participants completing 432 trials for each task.

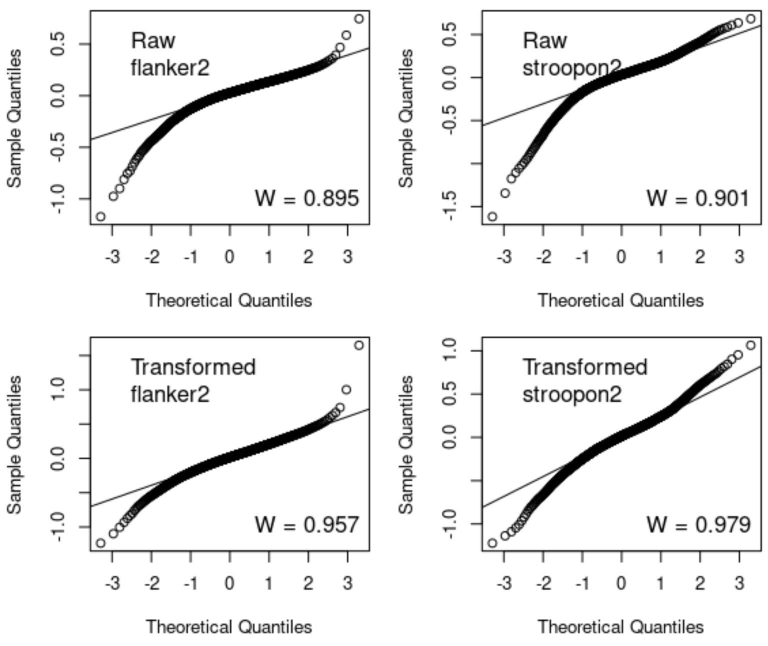

Supplementary Figure 2. Normality tests for the final non-gamified experiment. Q-Q plots and Shapiro-Wilk (*W*) values for each of the tasks. Top panel: Raw (untransformed) response time data, bottom panel: transformed ($\log_e$(RT-0.2)) response time data. The Q-Q plots on the transformed data indicate the data more closely fit a normal distribution. Combined with higher *W* values, this suggests that the transformation was effective. Based on participants completing 432 trials for each task.

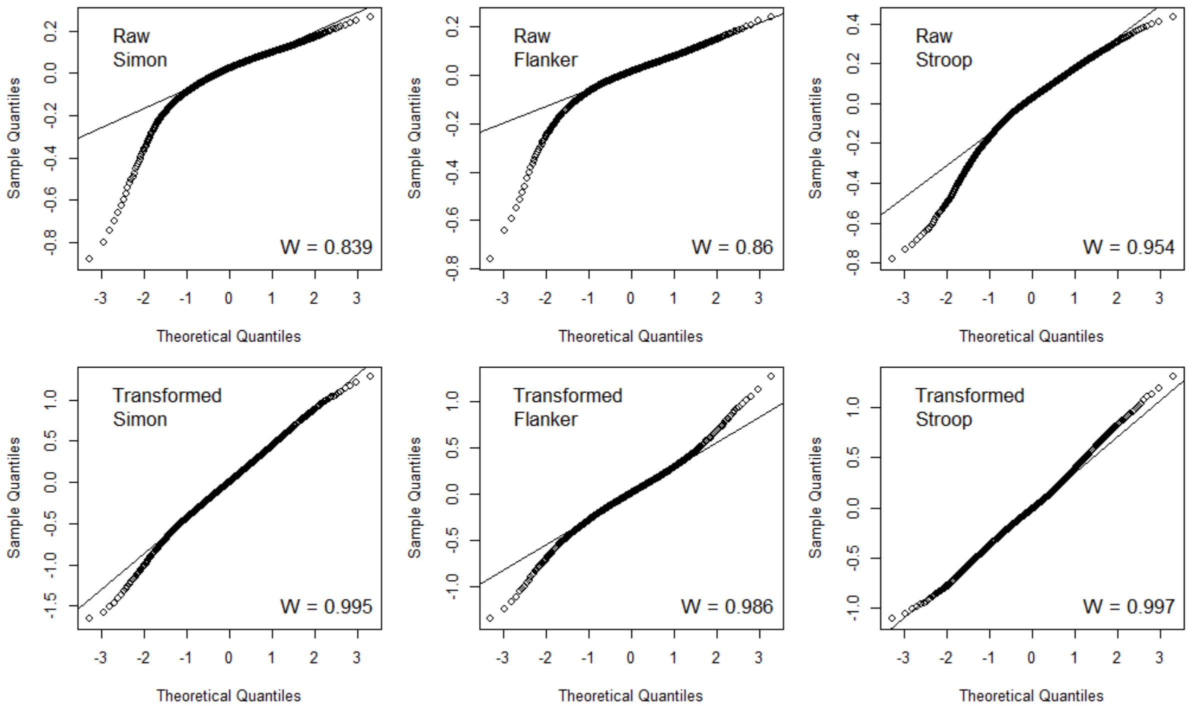

Supplementary Figure 3. Normality tests for Hedge et al.[1,2] data. Q-Q plots and Shapiro-Wilk (*W*) values for each of the tasks. Top panel: Raw (untransformed) response time data, bottom panel: transformed ($\log_e$(RT-0.2)) response time data. The Q-Q plots on the transformed data indicate the data more closely fit a normal distribution. Combined with higher *W* values, this suggests that the transformation was effective. Data from the first 432 trials for each participant per task.

Supplementary Table 1. Bayes Factors (BF) for the final gamified experiment.

Blocks	Flanker		Flanker2		Simon2		Stroop2		Stroopon		Stroopon2	
	BF1	BF2	BF1	BF2	BF1	BF2	BF1	BF2	BF1	BF2	BF1	BF2
1:2	3.45e+02	1.12e+01	1.22e-01	7.71e+00	3.55e+01	1.02e+01	3.53e+10	1.45e+01	1.02e+10	9.43e+00	2.25e+02	1.54e+01
1:3	3.78e+04	8.25e+01	1.47e+00	4.27e+01	3.96e+06	8.61e+01	3.53e+23	3.66e+00	3.44e+18	1.84e+01	2.80e+04	1.72e+02
1:4	7.56e+06	6.35e+02	1.24e+03	3.71e+02	3.22e+14	2.94e+02	2.54e+34	4.53e+01	1.97e+23	3.00e+01	9.37e+07	5.53e+02
1:5	2.76e+10	9.59e+02	1.45e+08	4.85e+00	3.34e+41	1.77e+03	5.34e+33	2.86e+02	4.61e+32	2.23e+02	2.96e+12	2.84e+03
1:6	9.09e+19	6.27e+03	1.43e+13	2.10e+01	7.41e+65	1.09e+04	6.14e+40	5.79e+02	1.44e+35	1.24e+03	4.29e+33	1.85e+04
1:7	1.17e+20	3.84e+04	4.08e+24	3.13e+01	7.44e+76	4.96e+04	3.26e+47	3.48e+03	6.14e+33	2.72e+03	3.82e+47	1.06e+05
1:8	4.02e+21	2.38e+04	5.59e+35	9.20e+01	7.43e+83	2.32e+05	5.69e+53	2.09e+02	3.85e+32	1.22e+04	3.32e+60	3.21e+05
1:9	3.72e+23	1.03e+05	7.13e+42	3.96e+02	7.88e+92	1.06e+06	3.54e+57	4.87e+02	9.02e+31	6.42e+04	3.25e+74	4.70e+05

Note. Bayes Factors reflect how many times more likely the observed data are under the assumed model compared to the alternative models. BF1 compares the standard model to a model assuming no practice effect. BF2 compares the standard model to a model assuming a practice × conflict effect interaction. Blocks are based on cumulative sets of 48 trials (i.e., the first row reflects 96 trials, and the final row reflects the full dataset of 432 trials).

Supplementary Table 2. Bayes Factors (BF) for Hedge et al.[1,2] data.

	Flanker		Simon		Stroop	
Blocks	BF1	BF2	BF1	BF2	BF1	BF2
1:2	4.20e-02	2.01e+01	2.83e+04	2.87e+01	7.23e-02	1.01e+01
1:3	3.13e-03	3.80e+01	3.55e+03	4.20e+02	1.84e-02	6.12e+01
1:4	3.83e+01	4.05e+02	1.31e+11	2.14e+00	1.34e+00	1.36e+02
1:5	3.36e+00	3.31e+03	1.08e+14	3.53e+01	1.44e-01	8.65e+02
1:6	2.61e+07	5.21e+02	3.95e+13	4.70e+02	1.36e-01	7.89e+03
1:7	5.42e+06	1.03e+03	3.05e+33	2.76e+03	1.42e-02	1.01e+03
1:8	1.95e+13	2.83e+01	2.83e+34	6.20e+03	3.95e-01	6.74e+03
1:9	9.15e+12	1.17e-01	3.15e+34	4.02e+04	3.54e+00	1.16e+03

Note. Bayes Factors reflect how many times more likely the observed data are under the assumed model compared to the alternative models. BF1 compares the standard model to a model assuming no practice effect. BF2 compares the standard model to a model assuming a practice × conflict effect interaction. Blocks are based on cumulative sets of 48 trials (i.e., the first row reflects 96 trials, and the final row reflects 432 trials).

Preliminary Experiments 1 and 2

Eight task variants were examined in Experiment 1: Flanker, Flanker2, Simon, Simon2, Stroopon, Stroopon2, Flankon, and Flankon2. See Methods for a description of the Flankon task; all other tasks remained consistent with those detailed in the main experiment. Experiment 2 consisted of Flanker2, Simon2, and Stroopon2.

Supplementary Results

Experiment 1

Results for each task in Experiment 1 are displayed in Supplementary Tables 3-10, where posterior samples were used to calculate median values and 95% credible intervals for the following: congruent and incongruent response times (RTs), the intercept (μ) and its standard deviation (SDμ), conflict effect (CE) and its standard deviation (SD_{id}), measurement noise (SD_n), trait precision (η), effect size (ES), and an estimation of the total number of congruent and incongruent trials required for adequate measurement (n).

We begin by looking at Flanker and Flanker2, where for both tasks, reliability ($r = .8$) was achieved within 27 trials given the total number of trials in the experiment (i.e., 48 per task). Flankon and Flankon2 also revealed promising results, achieving reliability in 26 and 38 trials, respectively. However, these complex variants showed no real benefit over the basic Flanker task in terms of reliability, although there was some indication of increased effect sizes. Contrasting with Flanker, the Simon task performed poorly, with 64 trials and 72 trials required to reach reliability of $r = .8$ for Simon and Simon2, respectively. That is, more trials than were presented would be necessary for adequate measurement in these tasks. Stroopon and Stroopon2 were placed between the aforementioned tasks, requiring 53 and 50 trials, respectively, to achieve reliability based on the 48 trials included in each task. In this instance, only a few more trials would be needed to result in reliable measurement. Both the conflict effect and η values were generally larger for the Flanker-based tasks (including Flankon) than the Simon tasks, with the exception of

Stroopon2. As a result, the tasks containing flanker required fewer trials to obtain reliable measurement. Regarding Simon and Simon2, the conflict effect was slightly larger in the latter, however, it produced marginally decreased reliability due to a larger increase in measurement noise than the individual differences in the conflict effect.

To add to these findings, Supplementary Fig. 4 provides the results for RT and accuracy for Experiment 1. In the Flanker variant of the tasks, the figures clearly demonstrate the present conflict effect that remained similar across all versions of the Flanker task, aside from some minor slowing in the Flankon task. Responses on incongruent trials were slower than responses on congruent trials. The larger effect for these tasks in comparison to the Simon-based tasks is also apparent. Again, there was some evidence of larger effects in the double shot conditions compared to the standard tasks. Additionally, despite not being the key variable of interest, we report that accuracy was superior in the congruent conditions compared to the incongruent conditions for all tasks.

In sum, Supplementary Fig. 4, and Tables 3-6, indicate that Flanker performed quite well on its own and when combined with Simon (i.e., Flankon), little additional benefit was found. Conversely, the Simon task alone was weaker, and reliability was improved when combined with the Stroop task (i.e., Stroopon2) as shown is Supplementary Fig. 4 and Tables 7-10.

Note that for each of the following tables, the rows give the 2.5%, 50% and 97.5% quantiles of the posterior values (i.e., the middle row is the median estimate, and the top and bottom rows give the associated 95% credible interval). Results are displayed in seconds.

Supplementary Table 3. Experiment 1 – Effect size and reliability analysis for Flanker

	congruent	incongruent	μ	SD_μ	CE	SD_{id}	SD_n	η	ES	ES_{id}	n
2.5%	0.660	0.794	0.790	0.280	0.129	0.137	0.269	0.438	1.050	1.192	37
50%	0.670	0.807	0.800	0.298	0.149	0.169	0.273	0.510	1.312	1.513	27
97.5%	0.680	0.820	0.810	0.317	0.170	0.206	0.278	0.578	1.585	1.870	21

Supplementary Table 4. Experiment 1 – Effect size and reliability analysis for Flanker2

	congruent	incongruent	μ	SD_μ	CE	SD_{id}	SD_n	η	ES	ES_{id}	n
2.5%	0.672	0.866	0.807	0.201	0.193	0.119	0.215	0.442	1.666	1.885	36
50%	0.681	0.879	0.816	0.213	0.211	0.141	0.217	0.512	1.960	2.258	27
97.5%	0.690	0.893	0.825	0.225	0.229	0.165	0.220	0.581	2.266	2.690	21

Supplementary Table 5. Experiment 1 – Effect size and reliability analysis for Flankon

	congruent	incongruent	μ	SD_μ	CE	SD_{id}	SD_n	η	ES	ES_{id}	n
2.5%	0.701	0.831	0.811	0.232	0.115	0.122	0.247	0.418	0.818	1.011	41
50%	0.716	0.851	0.825	0.257	0.144	0.153	0.251	0.519	1.070	1.375	26
97.5%	0.731	0.871	0.840	0.287	0.172	0.193	0.258	0.625	1.330	1.810	18

Supplementary Table 6. Experiment 1 – Effect size and reliability analysis for Flankon2

	congruent	incongruent	μ	SD_μ	CE	SD_{id}	SD_n	η	ES	ES_{id}	n
2.5%	0.754	0.956	0.897	0.219	0.186	0.126	0.270	0.342	1.356	1.754	61
50%	0.770	0.979	0.912	0.239	0.217	0.154	0.276	0.433	1.644	2.264	38
97.5%	0.786	1.001	0.927	0.261	0.247	0.187	0.283	0.532	1.943	2.953	25

Supplementary Table 7. Experiment 1 – Effect size and reliability analysis for Simon

	congruent	incongruent	μ	SD_μ	CE	SD_{id}	SD_n	η	ES	ES_{id}	n
2.5%	0.654	0.688	0.713	0.195	0.013	0.069	0.251	0.273	0.248	0.326	96
50%	0.664	0.699	0.722	0.206	0.030	0.086	0.254	0.333	0.479	0.640	64
97.5%	0.674	0.710	0.731	0.217	0.047	0.106	0.257	0.399	0.711	0.987	45

Supplementary Table 8. Experiment 1 – Effect size and reliability analysis for Simon2

	congruent	incongruent	μ	SD_μ	CE	SD_{id}	SD_n	η	ES	ES_{id}	n
2.5%	0.742	0.810	0.819	0.211	0.050	0.089	0.264	0.257	0.501	0.665	108
50%	0.755	0.825	0.830	0.225	0.072	0.109	0.266	0.315	0.737	1.008	72
97.5%	0.767	0.840	0.841	0.241	0.094	0.132	0.270	0.377	0.983	1.405	50

Supplementary Table 9. Experiment 1 – Effect size and reliability analysis for Stroopon

	congruent	incongruent	μ	SD_μ	CE	SD_{id}	SD_n	η	ES	ES_{id}	n
2.5%	0.638	0.717	0.737	0.244	0.063	0.099	0.241	0.288	0.627	0.890	86
50%	0.651	0.733	0.750	0.264	0.089	0.122	0.246	0.366	0.866	1.306	53
97.5%	0.665	0.750	0.763	0.285	0.115	0.153	0.252	0.454	1.100	1.806	35

Supplementary Table 10. Experiment 1 – Effect size and reliability analysis for Stroopon2

	congruent	incongruent	μ	SD_μ	CE	SD_{id}	SD_n	η	ES	ES_{id}	n
2.5%	0.921	1.065	1.058	0.28	0.107	0.182	0.400	0.303	0.545	0.771	78
50%	0.948	1.098	1.082	0.308	0.154	0.221	0.409	0.379	0.778	1.153	50
97.5%	0.975	1.132	1.106	0.338	0.203	0.267	0.418	0.469	1.006	1.594	32

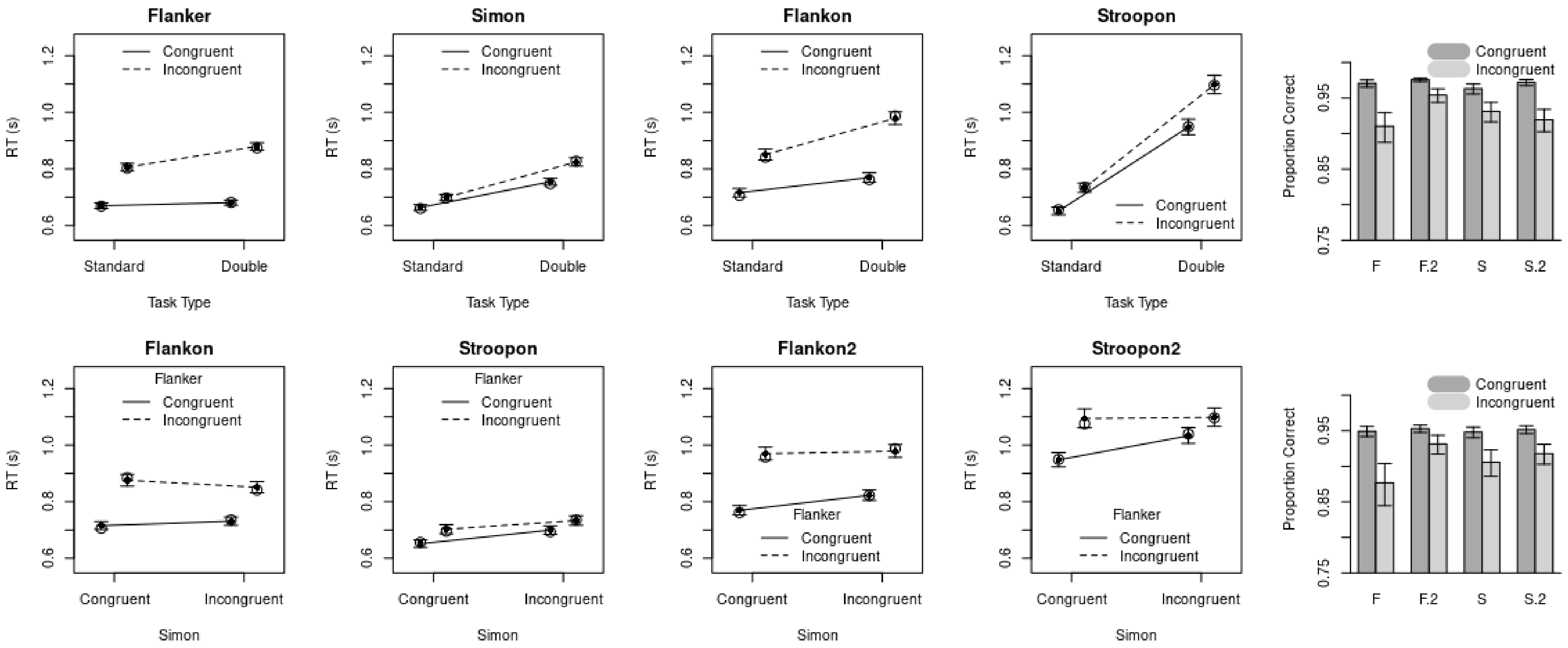

Supplementary Figure 4. Response time (RT) and accuracy for Experiment 1. RT data are displayed by open circles and the fit of the standard model (assuming practice effects) by lines joining solid points with 95% credible intervals. Top panel shows RT for congruent vs. incongruent trials in the standard tasks and the version with double shot trials. Accuracy (with 95% confidence intervals) is shown for Flanker, Flanker2, Simon, and Simon2. Bottom panel represents RT across each component for the combined tasks. The x-axis refers to congruent vs. incongruent trials for the Simon component of the combined tasks. Solid lines refer to congruent trials and dashed lines refer to incongruent trials for Flanker in the Flankon task or Stroop in the Stroopon task. Accuracy is shown for Flankon, Flankon2, Stroopon, and Stroopon2. Flanker, n = 80; Flanker2, n = 80; Flankon, n = 82; Flankon2, n = 82; Simon, n = 85; Simon2, n = 85; Stroopon, n = 88; Stroopon2, n = 88.

Experiment 2

As a result of the Flankon task failing to produce larger and more reliable conflict effects, we did not pursue it further. We proceeded to test Flanker2 alongside Simon2 and Stroopon2. Supplementary Tables 11-13 display the same information as the equivalent tables for Experiment 1. Again, Flanker2 was highly promising, with Simon2 performing poorest and Stroopon2 falling in the middle. Given the 48-trial block, reliability ($r = .8$) was achieved at 24 trials for Flanker2 and 47 trials for Stroopon2. Also similar to Experiment 1, Simon2 failed to reach reliable measurement and would instead require 66 trials (i.e., more than the number presented in this experiment). Both the conflict effect and trait precision (η) remained strong for the Flanker task and were relatively good for Stroopon.

In conjunction with the findings of Experiment 1, we found strong support for Flanker and Flanker2 and continued to use them in the final experiment. It also became apparent that Stroopon was the next best task and therefore both versions of the task were also used in the final experiment. We also included Simon2 and Stroop2 as the component parts of Stroopon2. Finally, the results of Experiment 2 suggested that always performing double shots trials was not advantageous, thus, we returned to implementing the double shot on 1/3 of trials.

Note that for each of the following tables, the rows give the 2.5%, 50% and 97.5% quantiles of the posterior values (i.e., the middle row is the median estimate, and the top and bottom rows give the associated 95% credible interval). Results are displayed in seconds.

Supplementary Table 11. Experiment 2 – Effect size and reliability analysis for Flanker2

	congruent	incongruent	μ	SD_μ	CE	SD_{id}	SD_n	η	ES	ES_{id}	n
2.5%	0.750	0.906	0.870	0.219	0.140	0.136	0.271	0.464	0.945	1.063	33
50%	0.763	0.922	0.881	0.235	0.164	0.170	0.274	0.539	1.218	1.388	24
97.5%	0.776	0.939	0.893	0.253	0.187	0.211	0.279	0.616	1.496	1.738	19

Supplementary Table 12. Experiment 2 – Effect size and reliability analysis for Simon2

	congruent	incongruent	μ	SD_μ	CE	SD_{id}	SD_n	η	ES	ES_{id}	n
2.5%	0.848	0.938	0.939	0.229	0.065	0.102	0.318	0.266	0.522	0.677	100
50%	0.864	0.957	0.953	0.248	0.093	0.126	0.322	0.329	0.785	1.056	66
97.5%	0.881	0.976	0.967	0.268	0.122	0.152	0.326	0.397	1.052	1.478	45

Supplementary Table 13. Experiment 2 – Effect size and reliability analysis for Stroopon2

	congruent	incongruent	μ	SD_μ	CE	SD_{id}	SD_n	η	ES	ES_{id}	n
2.5%	1.003	1.154	1.131	0.254	0.106	0.169	0.383	0.299	0.546	0.759	80
50%	1.033	1.190	1.157	0.289	0.157	0.208	0.391	0.388	0.811	1.183	47
97.5%	1.064	1.227	1.183	0.331	0.209	0.258	0.402	0.487	1.075	1.701	30

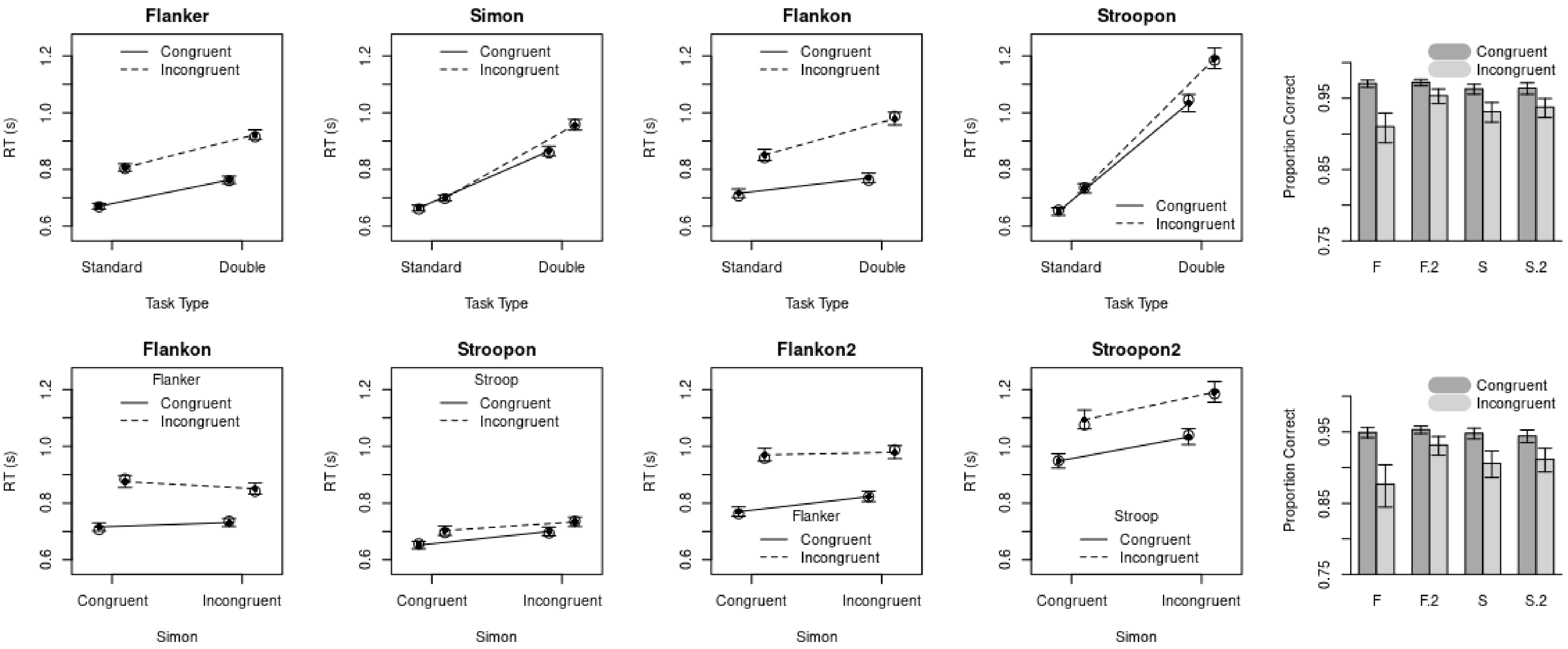

Supplementary Figure 5. Response time (RT) and accuracy for Experiment 2. RT data are displayed by open circles and the fit of the standard model (assuming practice effects) by lines joining solid points with 95% credible intervals. Top panel shows RT for congruent vs. incongruent trials in the standard tasks and the version with double shot trials. Bar graph shows accuracy (with 95% confidence intervals) for Flanker (F), Flanker2 (F.2), Simon (S), and Simon2 (S.2). Bottom panel represents RT across each component for the combined tasks. The x-axis refers to congruent vs. incongruent trials for the Simon component of the combined tasks. Solid lines refer to congruent trials and dashed lines refer to incongruent trials for Flanker in the Flankon task or Stroop in the Stroopon task. Bar graph shows accuracy (with 95% confidence intervals) for Flankon (F), Flankon2 (F.2), Stroopon (S), and Stroopon2 (S.2). The standard tasks and Flankon data are based on Experiment 1. Flanker, n = 80; Flanker2, n = 70; Flankon, n = 82; Flankon2, n = 82; Simon, n = 85; Simon2, n = 73; Stroopon, n = 88; Stroopon2, n = 70.

Supplementary Methods

Participants

In total, the sample for Experiment 1 comprised 1066 participants; we excluded 265 for failing the tutorial, 63 for exceeding the experiment's time limit, and 6 for incomplete data. A further 62 participants were excluded for low accuracy (< 60% overall), 5 for having too many anticipatory responses (> 10% of trials with RT < 0.1s), and 6 for non-responding (> 10% of trials not completed within 4 s). The final sample size was 670 across eight experimental conditions.

In experiment 2, a total of 394 participants were recruited. For failing the tutorial, exceeding the time limit, and incomplete data, we removed 144, 21, and 4 participants, respectively. Applying the same criteria as Experiment 1, we excluded the data of 2 participants for low accuracy, and 5 each for too many anticipatory responses and too many non-responses. This resulted in a final sample size of 213. Participants were required to have a human intelligence task (HIT; MTurk terminology for a task or study) approval rate of above 95%.

In both experiments, participants received a baseline payment of $1.00 USD for attempting the study. Passing the tutorial and completing the entire experiment resulted in a $0.50 bonus, with an additional bonus between $0 and $1.00 based on performance (i.e., up to $2.50 in total). Approval for this research was granted by the University of Tasmania's Human Research Ethics Committee.

Design and Materials

For Experiments 1 and 2, participants completed the tutorial and experimental phase in one session lasting approximately 15 minutes. For failing the tutorial, the total duration was approximately 10 minutes. Participants were randomly assigned to one of eight conditions for Experiment 1: (1) Flanker, (2) Flanker2, (3) Flankon, (4) Flankon2, (5) Simon, (6) Simon2, (7) Stroopon, (8) Stroopon2. And one of three conditions for Experiment 2: (1) Flanker2, (2) Simon2, (3) Stroopon2. The tasks and responding requirements of the various tasks was identical

to the final experiment (reported in the manuscript). An additional task, not described in the Method of the main text, was the Flankon task (see Supplementary Fig. 6 for illustrations). This task combines Flanker and Simon tasks, resulting in a similar display to that of flanker, however, the set of arrows is positioned to the right or left of screen to incorporate an element of a Simon task. The task remained the same as Flanker where the aim was to respond based on the central arrow while ignoring the flanking arrows and their location. For Flankon2, there were two types of second response required. A purple shield required a response based on the direction of the flanking arrows (left or right), while a yellow shield required a response dependent on the location of the arrows (left or right).

Procedure

For both Experiment 1 and Experiment 2, the eligibility criteria and tutorial procedure were consistent with the final experiment, as described in the main text. The experimental component consisted of 4 games (i.e., 48 trials in total) in a single session. Once finished, participants were advised of the bonus they received and were given an MTurk completion code.

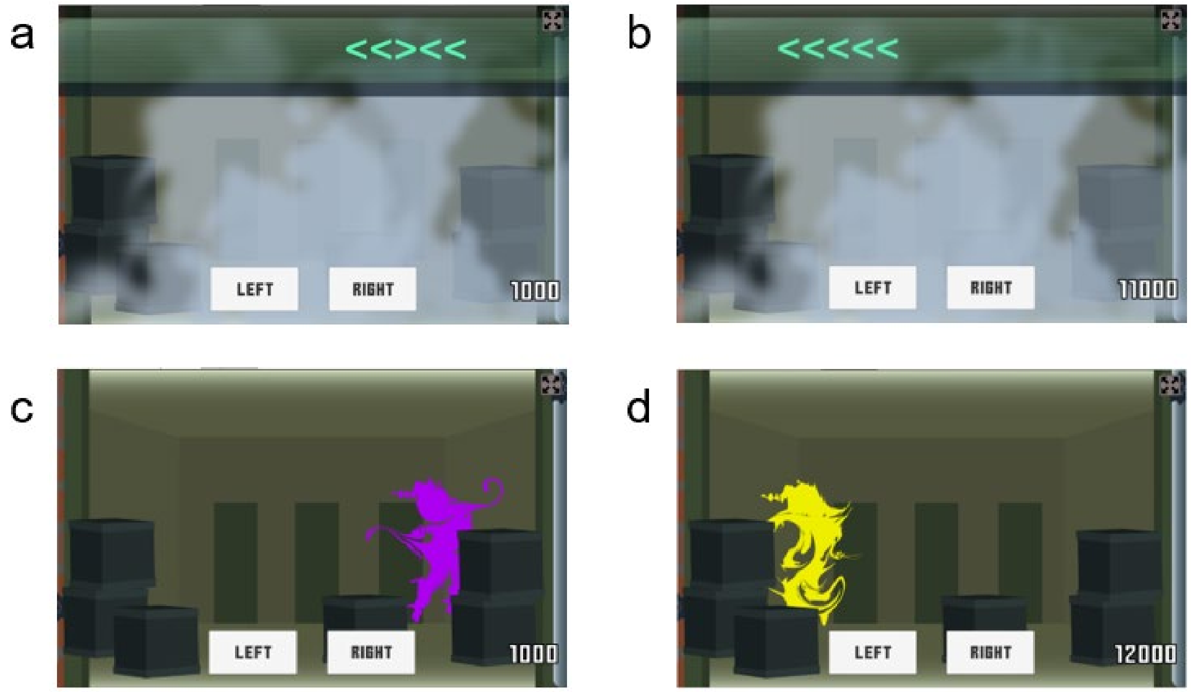

Supplementary Figure 6. Depiction of the Flankon task in Experiment 1. The task reflects a combined Flanker and Simon task. Responses are dependent on the central arrow as in the Flanker task with the set of arrows presented on the left or right of the display: a) incongruent Flankon display, and b) congruent Flankon display. After the initial response, an enemy may present with a shield, requiring a second shot to be made: c) second shot Flankon trial following a response to the display in (a), where a purple shield requires a decision based on the direction of the flanking arrows (i.e., left), and d) a second shot trial based on (b) requiring a decision based on location (i.e., left) in response to the yellow shield.

Supplementary References

1. Hedge, C., Powell, G., Bompas, A., Vivian-Griffiths, S. & Sumner, P. Low and variable correlation between reaction time costs and accuracy costs explained by accumulation models: meta-analysis and simulations. *Psychol. Bull.* **144**, 1200-1227 (2018).
2. Hedge, C., Powell, G. & Sumner, P. The reliability paradox: why robust cognitive tasks do not produce reliable individual differences. *Behav. Res. Methods* **50**, 1166-1186 (2018).
