# Supplementary material for: Calibration of cognitive tests to address the reliability paradox for decision-conflict tasks
Source: Nat Commun. 2023 Apr 19;14:2234. doi: 10.1038/s41467-023-37777-2 (PMC10115879; doi:10.1038/s41467-023-37777-2)
Supplement: Supplementary file 3 — Reporting Summary [file 41467_2023_37777_MOESM3_ESM.pdf]

nature portfolio

Corresponding author(s): Talira Kucina

Last updated by author(s): Feb 2, 2023

Reporting Summary

Nature Portfolio wishes to improve the reproducibility of the work that we publish. This form provides structure for consistency and transparency in reporting. For further information on Nature Portfolio policies, see our Editorial Policies and the Editorial Policy Checklist.

Statistics

For all statistical analyses, confirm that the following items are present in the figure legend, table legend, main text, or Methods section.

n/a	Confirmed	
☐	☒	The exact sample size (*n*) for each experimental group/condition, given as a discrete number and unit of measurement
☐	☒	A statement on whether measurements were taken from distinct samples or whether the same sample was measured repeatedly
☒	☐	The statistical test(s) used AND whether they are one- or two-sided<br>*Only common tests should be described solely by name; describe more complex techniques in the Methods section.*
☒	☐	A description of all covariates tested
☐	☒	A description of any assumptions or corrections, such as tests of normality and adjustment for multiple comparisons
☐	☒	A full description of the statistical parameters including central tendency (e.g. means) or other basic estimates (e.g. regression coefficient) AND variation (e.g. standard deviation) or associated estimates of uncertainty (e.g. confidence intervals)
☒	☐	For null hypothesis testing, the test statistic (e.g. *F*, *t*, *r*) with confidence intervals, effect sizes, degrees of freedom and *P* value noted<br>*Give P values as exact values whenever suitable.*
☐	☒	For Bayesian analysis, information on the choice of priors and Markov chain Monte Carlo settings
☐	☒	For hierarchical and complex designs, identification of the appropriate level for tests and full reporting of outcomes
☒	☐	Estimates of effect sizes (e.g. Cohen's *d*, Pearson's *r*), indicating how they were calculated

Our web collection on statistics for biologists contains articles on many of the points above.

Software and code

Policy information about availability of computer code

Data collection	Participants were recruited through Amazon Mechanical Turk (MTurk). Data collection, allocation of participants to experimental groups, and presentation of the experiment within a web browser was facilitated using the PlayUR website and API, which is custom code developed by L Wells. The Cogmission game presented within the web browser made use of the Unity game engine (commercial product, 2019.4.35f1), extended with custom code by L Wells.
Data analysis	Data were analysed using RStudio v1.1.383, R v3.6.0. Packages used were BayesFactor v0.9.12-4.2, coda v0.19-3, Matrix v1.3-2, stringr v1.4.0. Custom code was otherwise developed for data analysis.

For manuscripts utilizing custom algorithms or software that are central to the research but not yet described in published literature, software must be made available to editors and reviewers. We strongly encourage code deposition in a community repository (e.g. GitHub). See the Nature Portfolio guidelines for submitting code & software for further information.

Data

Policy information about availability of data

All manuscripts must include a data availability statement. This statement should provide the following information, where applicable:

- Accession codes, unique identifiers, or web links for publicly available datasets
- A description of any restrictions on data availability
- For clinical datasets or third party data, please ensure that the statement adheres to our policy

The data generated in this study have been deposited on OSF: https://osf.io/5f8tz/

The Hedge et al. data have been deposited on OSF: https://osf.io/cwzds/ (Stroop and Flanker tasks) and https://osf.io/btsrw/ (Simon Task). Access is also provided at the above OSF link.

Human research participants

Policy information about studies involving human research participants and Sex and Gender in Research.

Reporting on sex and gender	Information regarding sex and gender was not collected.
Population characteristics	See below.
Recruitment	Participants were recruited via MTurk and were required to have an approval rate of over 95%. They were able to view the study description before deciding whether to complete the study. As a result, self-selection bias may have occurred.
Ethics oversight	University of Tasmania - Human Research Ethics Committee.

Note that full information on the approval of the study protocol must also be provided in the manuscript.

Field-specific reporting

Please select the one below that is the best fit for your research. If you are not sure, read the appropriate sections before making your selection.

☐ Life sciences ☒ Behavioural & social sciences ☐ Ecological, evolutionary & environmental sciences

For a reference copy of the document with all sections, see nature.com/documents/nr-reporting-summary-flat.pdf

Behavioural & social sciences study design

All studies must disclose on these points even when the disclosure is negative.

Study description

This research reports on quantitative experiments where participants were randomly assigned to complete different versions of gamified conflict tasks (i.e., Stroop, Flanker, Simon and combined variants of these tasks) in an online setting.

Research sample

Participants were recruited through MTurk and were aged 18 years or over. Age (along with sex and gender) was not otherwise documented. Participants passed an English proficiency test and indicated they had normal or corrected-to-normal colour vision. The COVID-19 pandemic led us to collecting data from an online sample and benchmarking against archival data. Our rationale for this sample was that we were interested in testing the general population over 18 years of age and other demographic information was not relevant to the aims of this research (though participants were required to be fluent in English). Informed consent was obtained from all participants. Final sample size for each experiment is as follows: Main Experiment (gamified): N = 181, Main Experiment (non-gamified): N = 60, Experiment 1: N = 670, Experiment 2; N = 213.

Compensation (in USD): Main Experiment (gamified): $1.00 for failing tutorial, $10.00 for completing full study and a bonus of up to $7.20 was accrued based on performance. Main Experiment (non-gamified): $1.00 for failing tutorial, $16.00 for completing full study. Experiments 1 and 2: $1.00 for failing tutorial, $1.50 for completing full study and a bonus of up to $1.00 based on performance.

Archival data were reported by Hedge and colleagues in the following publications: Hedge, C., Powell, G., & Sumner, P. (2018). The reliability paradox: Why robust cognitive tasks do not produce reliable individual differences. Behavioral Research Methods, 50(3), 1166–1186. doi: 10.3758/s13428-017-0935-1; Hedge, C., Powell, G., Bompas, A., Vivian-Griffiths, S., & Sumner, P. (2018). Low and variable correlation between reaction time costs and accuracy costs explained by accumulation models: Meta-analysis and simulations. Psychological Bulletin, 144(11), 1200–1227. doi: 10.1037/bul0000164. We analysed data from the first publication for the Flanker and Stroop tasks, with a total of 112 (15 males) undergraduate student participants aged 18-47 years old. We analysed Simon data from the second publication, which involved 102 (12 male) undergraduate student participants. In both cases, participants were tested in person.

Sampling strategy	The main experiment was based on a series of three prior experiments. The preregistered sampling plan (https://osf.io/y4sbh) for the initial experiment used a sequential Bayes factor method, with a minimum sample size of 72 (for each task, i.e., Flanker task and Simon task) and a maximal sample size of 216. Data collection halted at the minimal sample size based on evidence favouring no difference between between experimental conditions.<br><br>For the proceeding two experiments, the basis of our sampling plan shifted to collecting enough participants to obtain reasonably precise standard errors on effect and reliability estimates, which we set at more than 70 participants per condition based on the results of the initial experiment.<br><br>The main experiment consisted of many more trials in each condition (i.e., 432 trials per participant) than the previous experiments. Given this increase and the attendant increase in measurement precision at the participant level, we reduced our sampling target to 30 participants per condition. This applied to all versions of the final experiment.
Data collection	Data collection was online via MTurk and as such participants completed the experiment on their own laptop or desktop computer. The Researchers were not blinded to the experimental conditions or hypotheses. However, participants were randomly allocated to an experimental condition upon agreeing to participate.
Timing	Main experiment: September-October 2021, follow-up experiment in July 2022 (Preliminary experiments: June-September 2021).
Data exclusions	Main experiment: 61 participants did not pass the tutorial, a test to ensure they understood the given task, and therefore did not proceed to the experimental phase. Another 9 were excluded due to incomplete data. Additionally, 3 were excluded for low accuracy (<60%), and one each for too many anticipatory responses (>10% of trials) and too many non-responses (>10% of trials). For the follow-up experiment, 14 failed the tutorial, 6 had incomplete data, and 1 was excluded for low accuracy.<br><br>Preliminary experiments: for Experiment 1 we excluded 265 participants for tutorial failures, 63 for exceeding the experiment's time limit, 6 for incomplete data, 62 for low accuracy, 5 for too many anticipatory responses, and 6 for too many non-responses. For Experiment 2 we excluded 144 participants for tutorial failures, 21 for exceeding the experiment's time limit, 4 for incomplete data, 2 for low accuracy, 5 for too many anticipatory responses, and 5 for too many non-responses.<br><br>These exclusion criteria were based on the criteria preregistered for the initial experiment in this series of experiments. Preregistration available on OSF: https://osf.io/y4sbh.
Non-participation	As data were collected online, participants opted into the research if they wanted to participate. In Experiments 1 and 2, there where 201 and 101 participants, respectively, who did not finish the experiment. For the Main Experiment, 44 participants did not complete the gamified version, and 17 did not complete the non-gamified version. This includes those who signed up but but did not start the experiment, as well as those who started but failed to complete the experiment in its entirety. Participants were free to withdraw at anytime, without explanation, therefore we do not have information regarding their reasoning.
Randomization	Participants were randomly allocated to an experimental group.

Reporting for specific materials, systems and methods

We require information from authors about some types of materials, experimental systems and methods used in many studies. Here, indicate whether each material, system or method listed is relevant to your study. If you are not sure if a list item applies to your research, read the appropriate section before selecting a response.

Materials & experimental systems

n/a	Involved in the study	
☒	☐	Antibodies
☒	☐	Eukaryotic cell lines
☒	☐	Palaeontology and archaeology
☒	☐	Animals and other organisms
☒	☐	Clinical data
☒	☐	Dual use research of concern

Methods

n/a	Involved in the study	
☒	☐	ChIP-seq
☒	☐	Flow cytometry
☒	☐	MRI-based neuroimaging
